# Real-time motion onset recognition for robot-assisted gait rehabilitation

**DOI:** 10.1186/s12984-022-00984-x

**Published:** 2022-01-28

**Authors:** Roushanak Haji Hassani, Mathias Bannwart, Marc Bolliger, Thomas Seel, Reinald Brunner, Georg Rauter

**Affiliations:** 1 BIROMED-Lab, Department of Biomedical Engineering, University of Basel, Basel, Switzerland; 2 Spinal Cord Injury Center, University Hospital Balgrist, Zurich, Switzerland; 3 Department Artificial Intelligence in Biomedical Engineering, Friedrich-Alexander-Universität Erlangen-Nürnberg, Erlangen, Germany; 4 University Childern’s Hospital Basel, Basel, Switzerland; 5 Sensory-Motor Systems Lab, D-HEST, ETH Zurich, Zurich, Switzerland; 6 Neuroscience Center Zurich (ZNZ), Zurich, Switzerland

**Keywords:** Real-time activity recognition, EtherCAT, Sliding window, Wireless EtherCAT interface, Inertial measurement unit, Body weight support

## Abstract

**Background:**

Many patients with neurological movement disorders fear to fall while performing postural transitions without assistance, which prevents them from participating in daily life. To overcome this limitation, multi-directional Body Weight Support (BWS) systems have been developed allowing them to perform training in a safe environment. In addition to overground walking, these innovative/novel systems can assist patients to train many more gait-related tasks needed for daily life under very realistic conditions. The necessary assistance during the users’ movements can be provided via task-dependent support designs. One remaining challenge is the manual switching between task-dependent supports. It is error-prone, cumbersome, distracts therapists and patients, and interrupts the training workflow. Hence, we propose a real-time motion onset recognition model that performs automatic support switching between standing-up and sitting-down transitions and other gait-related tasks (8 classes in total).

**Methods:**

To predict the onsets of the gait-related tasks, three Inertial Measurement Units (IMUs) were attached to the sternum and middle of outer thighs of 19 controls without neurological movement disorders and two individuals with incomplete Spinal Cord Injury (iSCI). The data of IMUs obtained from different gait tasks was sent synchronously to a real-time data acquisition system through a custom-made Bluetooth-EtherCAT gateway. In the first step, data was applied offline for training five different classifiers. The best classifier was chosen based on F1-score results of a Leave-One-Participant-Out Cross-Validation (LOPOCV), which is an unbiased way of testing. In a final step, the chosen classifier was tested in real time with an additional control participant to demonstrate feasibility for real-time classification.

**Results:**

Testing five different classifiers, the best performance was obtained in a single-layer neural network with 25 neurons. The F1-score of $86.83\% \pm 6.2\%$ and $92.01\%$ are achieved on testing using LOPOCV and test data ($30\%$, participants = 20), respectively. Furthermore, the results from the implemented real-time classifier were compared with the offline classifier and revealed nearly identical performance (difference = $0.08 \%$).

**Conclusions:**

A neural network classifier was trained for identifying the onset of gait-related tasks in real time. Test data showed convincing performance for offline and real-time classification. This demonstrates the feasibility and potential for implementing real-time onset recognition in rehabilitation devices in future.

**Supplementary Information:**

The online version contains supplementary material available at 10.1186/s12984-022-00984-x.

## Background

Spinal Cord Injury (SCI) leads to devastating consequences for the affected individuals. Due to the complete or incomplete disruption of the spinal cord, voluntary control and sensory function are diminished (incomplete SCI) or completely lost (complete SCI) below the level of the lesion [1]. This impairs or hinders the performance of daily activities like walking [2, 3]. Nevertheless, people with incomplete SCI can regain the ability to perform essential daily activities and enhance locomotor performance [4]. Through the recovery of mobility and improved performance of activities of daily living, quality of life also improves [5]. Mobility restoration can be strongly supported by intense rehabilitation training [6–8]. Hereby, patients should constantly face different challenging tasks like standing up/sitting down, overground walking, and stair climbing. Apart from stair climbing, standing up and sitting down are among the most demanding daily activities, even though they are a prerequisite to start other functional training tasks in daily life [9]. Several rehabilitation devices were designed to assist patients particularly during standing-up and sitting-down phases [10–13]. However, what is required is task-specific support and fall prevention in a way that the system prevents the patients from falling besides providing the needed amount of support while performing rehabilitation tasks [14]. If too much support is provided, the patients tend to become slack and do not actively train the rehabilitation tasks [15]. However, if too little support or even the wrong support is provided, the patients will not be able to complete or even initiate the desired task [15]. Being actively hindered or impeded during task execution can even demotivate patients to keep on trying. For instance, Body Weight Support (BWS) systems have been developed which provide a safe and permissive environment [16–18]. Unfortunately, most BWS systems can only support patients with unspecific vertical weight unloading which can lead, for example, to unphysiological standing up and sitting down transitions. A novel BWS system called “The FLOAT” (Reha-Stim Medtec AG, Germany) has been developed which can provide three-dimensional assistive forces and patient-specific body-weight support during walking [19]. The multi-directional BWS system “The FLOAT” can assist the patients while performing rehabilitation tasks by designing task-dependent support that works in harmony with the user’s movement. The designed controller can consist of haptic guidance along virtual elastic walls (e.g. based on passive potential fields), which guide the user’s movement [20–22]. In parallel, a force field in movement direction can assist the user’s motion while performing specific rehabilitation tasks such as sitting down, standing up, or walking. Since every rehabilitation task requires different support strategies, which need to be adjusted to the task, an algorithm is required in order to detect movement onset at an early phase, safely, robustly, and independently of the user in order to transition from one training task to another. For ideal support during specific rehabilitation tasks, task-dependent support should be selected and applied. Choosing the right support in an automated way for each task can be realized by detecting the onset and the type of the movements.

Onset recognition of standing-up and sitting-down motions for exoskeleton robots have been realized in various fashions. A simple and straightforward solution is that patients need to press a button that triggers support onset. This solution can be an unsafe and tiresome task for patients because pressing a button is an additional challenge for them [23]. Furthermore, one button usually is related to one task, consequently, triggering support for many specific tasks would require many buttons. Another solution for switching task-specific supports is using heuristic methods like threshold-based motion onset recognition that monitors, e.g. ankle and knee angles with potentiometers or other attached sensors to the rehabilitation device. This method is prone to false recognition due to the different threshold values that should be defined based on each subjet’s anthropometry [24, 25]. Unlike the solutions mentioned above, using wearable sensors like Inertial Measurement Units (IMU)s or Electromyography (EMG) sensors and applying machine-learning methods for activity recognition are gaining popularity among researchers in recent years [10, 24–31]. The approaches differ mainly in two points: (i) experimental setup, (ii) recognition methods.

**Fig. 1:**
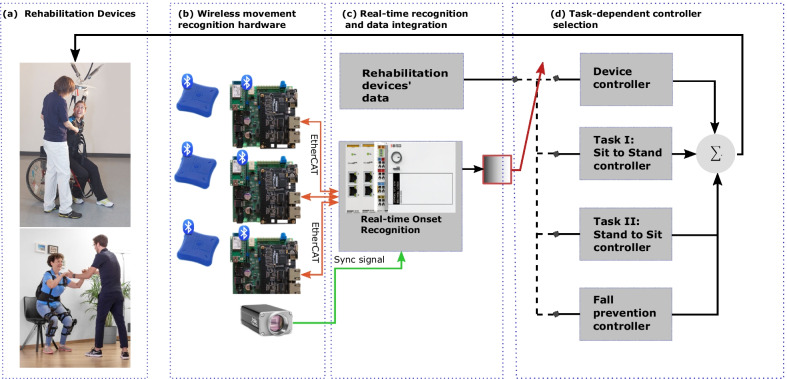
Implementation of a real-time motion onset recognition framework for rehabilitation devices. **a** Rehabilitation devices: The FLOAT(Reha-Stim Medtec AG, Germany), Myosuit (MyoSwiss AG, Switzerland). **b** Wireless interface boards for acquiring data from wireless IMUs. **c** Synchronized data acquisition from rehabilitation devices, sensors, and real-time movement onset recognition. **d** Selection of task-dependent supports (Sit-to-Stand controller/Stand-to-Sit controller/device Controller) based on the recognized task (Sitting down/Standing up/other activities)

(i) The experimental setup refers to the number of sensors and sensor placement, which varies based on the specific application and how fast the activity should be recognized [32]. For instance, although attaching sensors to the lower body shows good accuracy in recognizing sitting-down and standing-up onsets [25], it detects motion onset late because sitting down and standing up are initiated by a movement in the upper body [10].
(ii) Developing a recognition method for detecting the onset of the movements also highly depends on the application: online or offline recognition. Machine learning as an offline recognition approach has shown robust performance on pre-segmented sequences of activities in control participants [33]. However, real-world applications require online activity recognition on streamed unprocessed data that comes without pre-segmentation [34]. Besides, when performing recognition online on streamed data, the developed recognition model needs to be executable in real time without substantial delay. Therefore, in addition to accuracy, also feasibility, and speed of the classification should be considered for real-time applications. Moreover, for including movement onset recognition in combination with robotic devices that provide support according to the results of the recognition, some prerequisites need to be met. Unlike traditional data acquisition systems that used the point-to-point connection structure between the PC and sensors [35], many rehabilitation tasks need the involvement of many types of sensors with different sampling rates and communication protocols. The requirement for using many data acquisition systems at once makes the data acquisition and processing highly challenging, and asynchronous data leads to drifts in the time scales of the different acquisition systems. Therefore, a new protocol for the synchronized transmission of large data packages has been established: EtherCAT. EtherCAT is the modified version of Ethernet from EtherCAT.org to address the concern of synchronized data transmission at high speed and reliability [36]. In this paper, we propose real-time motion onset recognition with IMUs, which are attached to the human body. Figure 1 depicts the long-term goal for real-time motion onset recognition. The motion onset recognition model provides the decision which controller needs to be applied for providing ideal support for the task that is currently performed. The herein provided algorithm can be applied to various rehabilitation devices like BWS systems/exoskeletons. In this work, first, the design and implementation of an EtherCAT interface for wireless data acquisition is presented that transfers data from IMUs via Bluetooth protocol into the real-time data acquisition system using the EtherCAT data transmission protocol (Fig. 1b). Second, eight distinct tasks are recognized in real time with machine learning techniques. The best model out of 5 recognition models implemented with different machine learning models is selected. The best recognition model can then be used in a real-time implementation to select the appropriate task-dependent controller (Fig. 1c). For instance, the controller of “The FLOAT” will switch to “stand-to-sit” controller in case onset of sitting down is recognised (Fig. 1d).

## Methods

### Hardware and firmware development

**Fig. 2:**
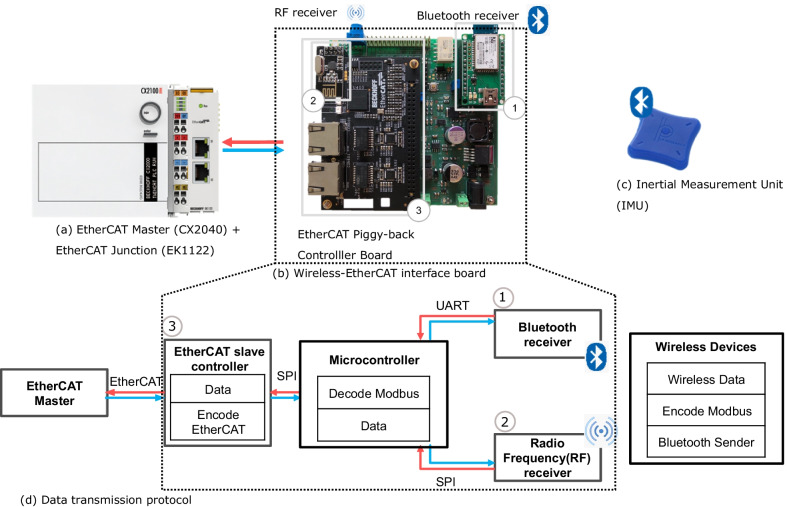
Data acquisition system components with corresponding data flow: **a** Real-time embedded PC (EtherCAT Master), **b** Wireless-EtherCAT interface board, **c** Inertial Measurement Unit (IMU), **d** Data transmission protocol from wireless devices to real-time embedded PC (EtherCAT Master)

A synchronized data acquisition (DAQ) system was used for reliable data transmission and acquisition. This DAQ consists of an embedded PC (CX2040, Beckhoff Automation Gmbh, Verl, Germany) running a real-time operating system (Fig. 2a). Communication and data exchange between the real-time operating system and the inputs and outputs (slaves) took place over EtherCAT protocol (real-time Ethernet) allowing all input tasks to be triggered and output tasks to be updated via a common pulse to ensure proper synchronization.

Despite the advances in wireless data transmission, power management, and small wireless wearable sensors, there is no commercial EtherCAT slave available to acquire data into real-time embedded PCs from wireless sensors. For this reason, we developed an EtherCAT gateway board enabling wireless data acquisition (Fig. 2b). Figure 2d shows the structure of the data transmission protocol from wireless devices to the embedded PC. The board received data which was encoded with Modbus protocol via a Bluetooth $\textcircled {1}$ or radio frequency (RF) $\textcircled {2}$ receiver module. Received data was transferred to a 32-bit micro-controller (PIC32mx4704f512l, Microchip Technology Inc., USA) via Serial Peripheral Interface (SPI) or Universal Asynchronous Receiver-Transmitter (UART) protocol. The microcontroller was programmed using the EtherCAT Slave Stack Code tool (EtherCAT Technology Group, Nürnberg, Germany) to encode the EtherCAT protocol with the decoded data from the Modbus protocol. Data was then ready to be transferred via EtherCAT protocol to a synchronized real-time data acquisition system. Data transformation to the real-time system (EtherCAT Master) was performed with the EtherCAT Piggyback Controller board $\textcircled {3}$ FB1111-0142 (Beckhoff Automation GmbH Co. KG, Germany). The EtherCAT Piggyback controller board combined an ET1100 EtherCAT Slave Controller, two EtherCAT ports, and a PDI-connector on one printed circuit board that was mounted onto a custom-made wireless data acquisition board. The EtherCAT Piggyback controller board was coupled with the embedded PC using a 2-port EtherCAT junction (EK1122, Beckhoff Automation GmbH Co. KG, Germany).

### Measurement setup

**Fig. 3:**
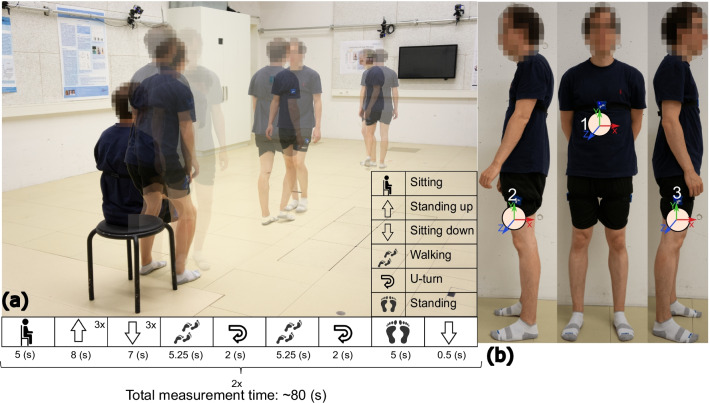
Data acquisition and sensor placements: **a** Measurement protocol (starts from sitting position for a few seconds followed by standing up and sitting down three times (3×) then, walking and turning, standing without any motion for few seconds, and sitting). **b** Sensory set-up (one IMU on the sternum and two IMUS on the middle of outer thighs)

The above developed setup for deterministic and synchronized data acquisition formed the basis of receiving and sending wireless real-time data. Three wireless IMUs (LPMS-B2, LP-RESEARCH Inc., Tokyo, Japan) were attached to the sternum $\textcircled {1}$ and the middle of both outer thighs $\textcircled {2}$ $\textcircled {3}$ of each participant (details on the axes orientation are presented in Fig. 3b). The LPMS-B2 is an IMU with an integrated 3D accelerometer, 3D gyroscope, and 3D magnetometer. Data output format from the IMU could be the sensor’s raw data, Euler angles, and quaternions. Data was broadcasted via Bluetooth 2.1 + EDR/Low Energy (LE) 4.1 and could be received in distances up to 20 m. In this work, raw data from the accelerometer and the gyroscope sensors were streamed via Bluetooth and received with the custom-made board (see subsection ‘Hardware and firmware development’). This board was used as a gateway for acquiring data in the real-time setup. Data was continuously collected with a sampling rate of 100 Hz. Furthermore, the performed tasks were recorded via a video recording system (Basler piA640-210gc, Basler AG, Germany) at 50 fps sampling rate synchronously for labelling the different recorded movements.

### Participants

To recognize the movement onset of different gait-related activities, a study was conducted. The study was approved by the local ethics committee of the Canton of Zurich, Switzerland (BASEC-Nr. 2016-0193) and conducted in accordance with the Declaration of Helsinki. The 19 control participants without neurological movement disorder (9 females) and 2 individuals with iSCI (1 female), (lesion level T10 and C6, respectively, and both ASIA D) were recruited for data collection. The average height, weight, and age of the participants were $173.14\pm 10.77$ cm, $66.57\pm 12.07$ kg, and $28.95\pm 4.5$ years, respectively.

### Study protocol

A study protocol to acquire data from participants was designed as follows: 3 IMUs were attached to the sternum and the middle of both outer thighs of each participant (see Fig. 3). The participants were asked to perform several repetitions of “Sit to Stand” and “Stand to Sit” activities and four other movements: sitting, standing, and walking with U-turns at their self-selected speed. The measurement protocol started from sitting without moving, followed by standing up and sitting down three times, walking, making a right- or left-directed U-turn, walking back to the chair position, making another right- or left-directed U-turn, standing without moving for a few seconds, and sitting down. This procedure was repeated twice.

The total number of repetitions for sitting and standing tasks for each participant was 6, while the total number of repetitions was 4 for walking and turning. The average time of measurement was 80 s and 100 s for control and iSCIs, respectively (see Fig. 3a).

Collected data from the IMUs was sent to the real-time data acquisition system, through the self-developed Bluetooth interface boards and was logged on the EtherCAT master. Logged data was used for generating an offline classification model to recognize the onset of the performed tasks. In order to provide extra information to the assistive device, some tasks like sitting down and standing were segmented into two phases [10]. Therefore, in total eight classes of activities were recognized, namely:

- *Sit to Stand* (Movement starts from bending the upper body and ends by leaving the chair)
- *M. Standing* (Motion of Standing: movement starts from leaving the chair and ends when a stable, motionless standing posture is reached)
- *Stand to Sit* (Movement starts from the bending upper body and ends by touching the chair)
- *M. Sitting* (Motion of Sitting: movement starts when contacting the chair and ends when a stable, motionless sitting posture is reached.
- *Walking* (Walking straight with self-selected speed)
- *Turning* (Making a $180^{\circ }$ turn with self-selected directions (left/right))
- *Standing* (Standing without motion)
- *Sitting* (Sitting without motion)

### Offline model generation

For generating a movement onset recognition model, which was used later in real-time motion onset recognition, first, 5 different classification models were trained, tested, and compared for their offline performance. To evaluate the performance of each classifier Leave-One-Participant-Out Cross-Validation (LOPOCV) [10] was conducted for each classification method (18 controls, 2 individuals with iSCI). Such that each classification model trained and tested 20 times. Iteratively, data of 19 participants were used as a train data set to train the model and data of 1 participant was used to test and evaluate the performance (data of each participant was used 19 times in the train data set and once in the test data set). The average of the F1-scores is reported to compare the performance.

**Fig. 4:**
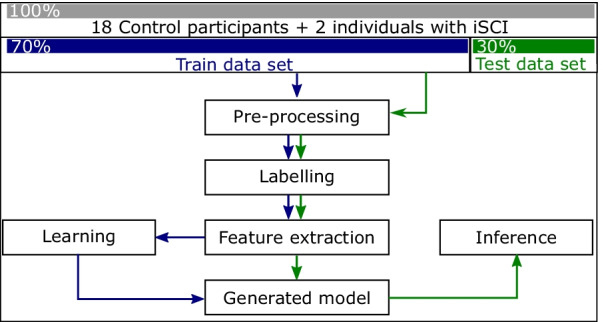
Train and test data preparation and recognition model generation workflow

Subsequently, data from 18 out of 19 controls and 2 individuals with iSCI was divided randomly into training (*Training data set*) and test (*Test data set*) data: 70% and 30%, respectively. The random division was selected in a way so that all activities were represented equally in train and test data sets (see Fig. 4). The training data was used to develop a final recognition model to recognize the onset of the introduced activities in subsection ‘Study protocol’. The last participant was later used in a real-time scenario to check the performance of the final recognition model.

The data preparation procedure was the same for the training and test data: starting from signal pre-processing, followed by labelling, and feature extraction. Extracted features from the training data and the corresponding label for each feature set were used in the learning block for model generation. Consequently, the generated model was examined with extracted features from the test data set in the inference block. Finally, predicted labels were compared with actual labels to check the performance of the inference block (see Fig. 4). The following subsections describe each step in detail.

#### Signal pre-processing and labelling

The first step after acquiring data was signal pre-processing, in which the signal bias of angular velocity and acceleration were removed, and angular velocity signals were scaled between $-1$ and 1 to avoid a broad range of values in the features. Acceleration signals were used without processing since scaling and filtering did not improve the classification results. Furthermore, the orientation of the IMUs were estimated by employing a quaternion-based sensor fusion algorithm that used strap-down integration of the angular rates and geodetic accelerometer-based drift compensation [37]. The magnetometer readings were not used in orientation estimation due to hard and soft iron disturbances on the magnetometer data in indoor environments [38]. Consequently, manual labelling of the signals based on videos that were captured synchronously with the IMU sensors during measurements was conducted. Time-stamps for different activities based on the videos were extracted. Then, the corresponding parts of the signals from the acquired data were labelled.

#### Feature extraction

The classification of a dynamic activity requires a certain limited amount of data history to obtain a reasonable estimation of the activity. The reason is that one single sample from one specific time instant cannot fully represent the performed activity. Thus, the entire set of streamed signals was divided into windows of equal size with constant overlapping time. Hereby, finding an optimal window size was critical because if the chosen window was too small, it might not contain enough information to represent the particular activity. On the contrary, if a too wide window was chosen, this window might contain information from two or more classes of activity as well as introduce delays in real-time recognition.

**Fig. 5:**
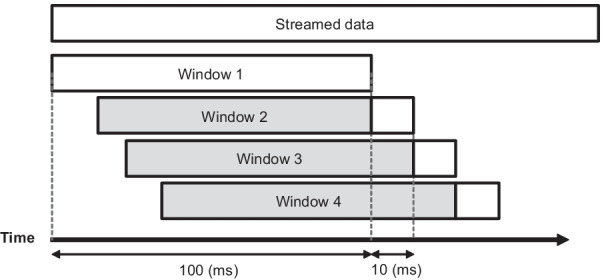
Moving window with 100 ms length and 90% overlap

Therefore, to find an optimal window size for feature extraction, the time of executing different activities for each subject was calculated, and the shortest execution time was identified across all participants. Considering that the shortest activity time occurs in 200 ms for some of the participants, the chosen window size should be less than 200 ms to not miss any activity and not have a big overlap with other activities. Thus, the final window size was set to 100 ms with a 90% overlap for handling the transitions between different activities and to shorten the classification delay in real-time (Fig. 5).

For each window, 55 time-domain features such as statistical values, a derivative of a fitted line to angular rates, accelerations, and estimated orientations were extracted (see Additional file 1: Table S1). Then, the extracted features were ranked and cross-validated by the recursive feature elimination method provided in [39]. A subset of features was selected based on recursive feature elimination using SVM estimators by removing 0–55 features. Then, 27 features (the best subset of features) were selected based on the cross-validation score of the model. These were the 27 features that were used for all recognition models. The selected features were scaled employing min–max scaling before training of the recognition model. In parallel, signals were manually labelled using the synchronously captured videos. Each moving window was labelled using a majority voting criterion, meaning if one task was the dominant task in a time window, then the window was labeled with the label of the dominant task. Each labelled window was combined with the generated feature vector to construct a training vector. Moreover, to have a balanced number of samples across tasks in the training data set, tasks with higher number of samples were down-sampled.

#### Learning and inference

Many supervised classification algorithms have been employed in human activity recognition. The algorithms differ from several points of view, such as the feature set, the number of classified activities, computational cost, calculational speed, memory usage, and classification accuracy [39, 40]. Therefore, common classification methods in human activity recognition were employed for learning and inference to choose the best technique suitable for real-time applications.

The training vector was applied to train five classification methods: decision trees [41], K-Nearest Neighbour (K-NN) [42, 43], Support Vector Machines (SVM) [43], Linear logistic regression [44], and Neural Networks [45]. The classification method was not only chosen based on a high F1-score in LOPOCV, but it should also be implementable and executable in a real-time setup. Accordingly, to assess the potential of the different classifiers for a real-time implementation, also the prediction speed in of each classifier was investigated on an Intel(R) Core(TM) i$7-8565$U CPU @ 1.80 GHz computer (see Table 1). Additionally, LOPOCV allows detailed insight into the variability of the classification accuracy for individual participants, which provides deeper insight into the robustness of the classifier for new data due to unbiased testing. To obtain a final model and also to compare unbiased vs. biased testing data sets, $70\%$ of data obtained from the 20 participants was taken. The final model was then tested with the remaining $30\%$ of the 20 participants. For both testing and training scenarios using the 20 participants (i) LOPOCV and (ii) $70\%$ training and $30\%$ testing data, classification results are represented in form of confusion matrices and the F1-score. The confusion matrix reports the number of false-positive (incorrectly identified), false-negative (incorrectly rejected), true-positive (correctly identified), and true-negatives (correctly rejected) observations. These factors allow us to perform a more detailed analysis of the results like precision, sensitivity, specificity, and F1-score for each class rather than basing our decision only on overall accuracy and error rate. The definition of each expression is presented in the following.

**Table 1:** Comparison between classification methods for eight classes of activities

Classifier	Prediction speed (obs^a^/s)	F1-score (%) (LOPOCV)
Decision tree	4,006,000	$78.87 \pm 11.6$
KNN (neighbors = 3)	10,060	$78.34\pm 8.4$
Linear SVM	5,120,000	$85.40 \pm 5.07$
Linear logistic regression	4,067,000	$83.9\pm 6.4$
Neural Network	2,268,000	$86.83\pm 6.2$

^a^Observation

*Precision* indicates the proportion of true-positives over the number of true-positives plus false-positives.

*Sensitivity* indicates the proportion of true-positives over the number of true-positives plus the number of false-negatives.

*Specificity* indicates the proportion of true-negatives over the number of true-negatives plus false-positives.

*F1-score *indicates the harmonic mean of precision and sensitivity.

### Real-time onset recognition

In order to provide robotic support during different activities with rehabilitation devices like BWS systems/exoskeletons, the onset of activities should be recognized in real time to provide an input signal for the device that will trigger the switch between task-dependent supports. Similar to offline classification, the workflow for real-time classification started with data acquisition from the sensors, followed by pre-processing (scaling and offset removal). However, the difference to the previously explained offline process was that the data processing was performed on the fly on the incoming data set. The best offline classification model according to our criteria (see subsection ‘Offline model generation’), was implemented in Simulink 2017b. Then C++ code was generated to obtain a program that can be further compiled to machine code and executed in real time on the embedded PC. Figure 8 depicts a real-time recognition timing diagram in the embedded PC. Data acquisition and pre-processing were executed in one real-time cycle, which was 2 ms. To extract features from streaming signals in real time, circular buffers were implemented to enable 100 ms of overlap of the streamed signals every 10 ms. This means that every 10 ms features were extracted from each segmented window as soon as the desired buffer was filled. Then, the features were fed to the neural network model for activity recognition. Furthermore, each milestone of the workflow was logged for validation of real-time classification later on.

## Results

### Offline classification

Using the recursive feature elimination method, 27 dominant features were selected from 55 extracted features. The selected features that were used for training the prediction models are presented in Additional file 1: Table S1. In Table 1, results from the evaluation of different classification methods are presented.

Statistical evaluation of the model choice based on the accuracy expressed in F1-score using One-way ANOVA revealed a statistically significant effect $F_{4,95}=6.07, p<0.0005$ (significance level $p=0.05$). Pair-wise comparison using the Tukey-Kramer test, revealed the following results, which will be presented in the following way: advantage model 1 over model 2, p-value: e.g. m1 > m2, $p=0.023$. The following model abbreviations are used: Decision Tree (DT), K-Nearest Neighbours (KNN), Support Vector Machine (SVM), Linear Logistic Regression (LLR), Neural Network (NN): KNN > DT, $p=1.0$; SVM > DT, $p=0.0324$; LLR > DT, $p=0.0264$; NN > DT, $p=0.0041$; SVM > KNN, $p=0.0434$; LLR > KNN, $p=0.0356$; NN > KNN, $p=0.0058$; LLR > SVM, $p=1.0$; NN > SVM, $p=0.9589$; NN > LLR, $p=0.9728$. Accordingly, the SVM, LLR, and NN perform significantly better than the DT and the KNN, but there is no statistical difference between the SVM, LLR, and NN. A qualitative comparison of results showed that the mean F1-score for the LOPOCV is highest for the Neural Network model while the standard deviation is comparably low and prediction speed is relatively high. Therefore, the Neural Network model was chosen for further consideration.

Within the Neural Network, a Competitive Soft Transfer Function and a Sigmoid Symmetric Transfer Function were implemented in Matlab and used as activation functions for hidden layer and output layer, respectively. The “Scaled Conjugate Gradient” (trainscg) is used as the training function. The number of neurons in the hidden layer was searched and selected in the range of 15–27 neurons using grid search for different window sizes and overlaps. 25 neurons in the hidden layer were found to be the best trade-off between the window size of 100 ms and an overlap of 90% for model responsiveness (see Additional file 1: Table S2).

**Fig. 6:**
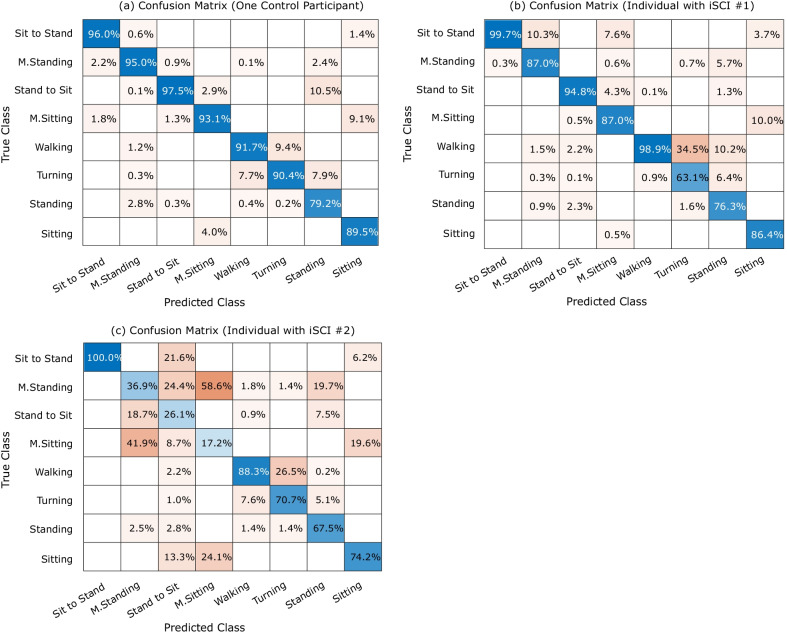
Leave-One-Participant-Out Cross-Validation (LOPOCV) results on **a** Random control participant (F1-score: $93.55\%$), **b** Individual with iSCI #1 (F1-score: $85.13\%$ ), **c** Individual with iSCI #2 (F1-score: $66.41\%$)

Confusion matrices from LOPOCV for one control participant and the two individuals with iSCI are presented in Fig. 6. To exemplify the performance of the classifier in controls and individuals with iSCI, a comprehensive overview of results from LOPOCV can be found in the supplementary material (see Additional file 1: Fig. S1).

**Fig. 7:**
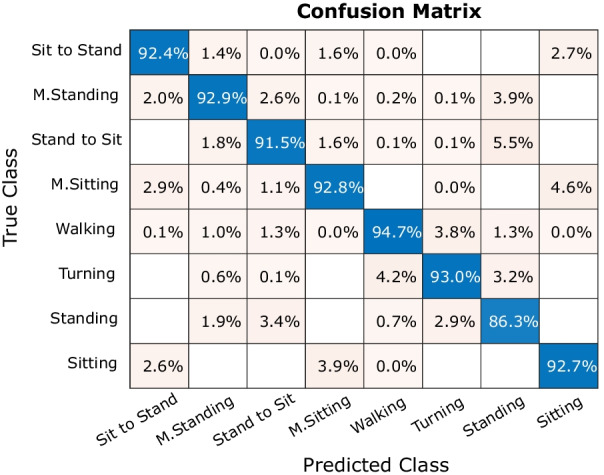
Offline model performance of the Neural Network on the test data set: confusion matrix for eight classes of activities (overall accuracy: 92.01%)

Furthermore, classification results for eight classes of activity on the test data set are shown by the confusion matrix in Fig. 7. Each row of the confusion matrix shows the actual (i.e., true) class, and each column presents the predicted class. Blue cells indicate the percentage of accurately classified observations (i.e. true positives), and yellow cells show the percentage of false recognitions. The overall accuracy for recognizing eight classes of activity in the test data set was 92.01%.

**Fig. 8:**
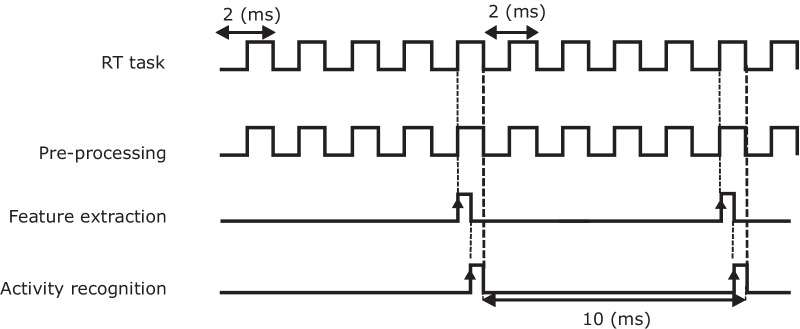
Real-time recognition timing diagram: inputs from sensors updated and processed every 2 ms, buffered for 10 ms, and activity classified every 10 ms

The evaluation report in Table 2 depicts that the classification using the test data set achieved an accuracy and specificity for each class of activity higher than $97\%$ while the precision and sensitivity of some classes of activity are lower (Fig. 8).

**Table 2:** Evaluation report for offline classification of eight activities (using Neural network model) on the test data set

Activity class	Accuracy (%)	Precision (%)	Sensitivity (%)	Specificity (%)	F1-score (%)
Sit to stand	98.49	92.43	89.58	99.32	90.98
M.standing	98.03	92.88	90.92	99.02	91.89
Stand to sit	97.73	91.48	89.39	98.86	90.42
M.sitting	97.80	92.80	90.89	98.89	91.83
Walking	98.19	94.70	94.58	98.92	94.64
Turning	97.92	93.00	89.51	99.07	91.22
Standing	96.87	86.25	92.23	97.62	89.14
Sitting	97.97	92.69	95.77	98.43	94.21

### Real-time classification

**Fig. 9:**
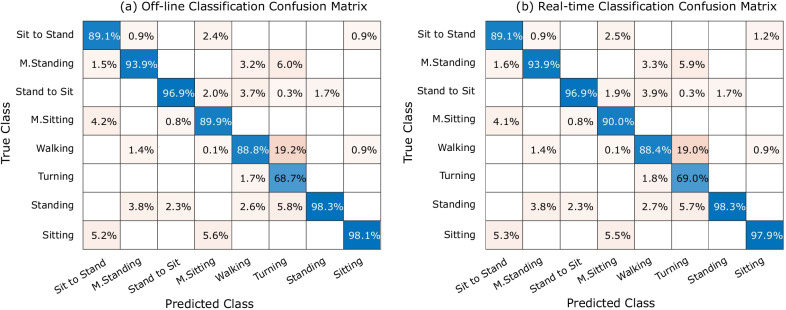
Offline versus real-time classification: **a** Confusion matrix for offline classification (F1-score: 88.68%), **b** Confusion matrix for real-time classification (F1-score: 88.60%)

**Fig. 10:**
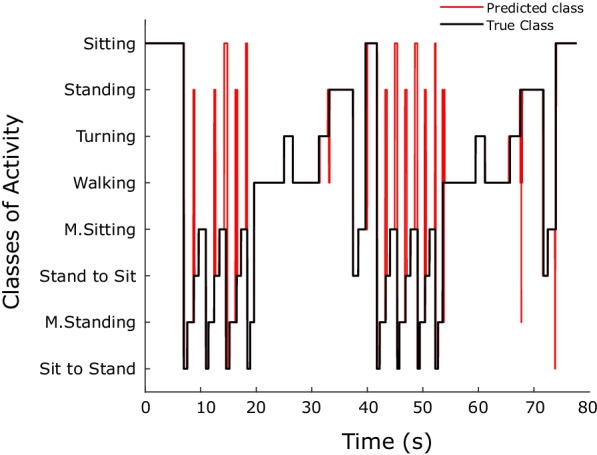
Onset recognition results for a representative subject. Black line presents “True class” and red line shows the “Predicted class”. False classification occurs only shortly and mostly near transitions between classes

In order to assess the implemented real-time onset recognition algorithm, data from a control subject was acquired, and the activities were classified in real time. For validation, labels from the real-time classification were compared with the offline classification results by using data that had been logged in parallel to the real-time classification from the sensors during real-time measurement. Figure 9a and b present the confusion matrices for offline and real-time classification, respectively. The generated offline classification method was examined on one participant that was not in test and training data from before. The F1-score of $88.68\%$ and $88.60\%$ were obtained for the offline and real-time classification, respectively. Figure 10 illustrates the recognition of streamed data for the same subject. The *x*-axis shows the time of the measurement, and the *y*-axis shows the gait-related tasks. Predicted class and true class have been shown with red and black, respectively.

## Discussion

This paper presents real-time motion onset recognition for different gait-related tasks using machine learning techniques. While classification of different gait-related tasks is of high interest for automated gait analysis, we go one step further and show feasibility of gait-related task classification in real-time. In particular, we are interested in real-time motion onset recognition for switching between task-dependent supports during the respective tasks with rehabilitation robots. Exemplarily, by recognizing the initiation of sitting down and standing up, task-dependent supports can be switched automatically.

The onset recognition and switching between supports needs to be performed in a deterministic way that will allow synchronized real-time control of the rehabilitation device as well as real-time data acquisition from sensors (IMUs). In order to enable synchronized real-time data acquisition of several Bluetooth-based commercial IMUs, a wireless interface board was designed that transfers data to the real-time system (Embedded PC). Three IMUs have been used: one was attached to the sternum and two were attached to each thigh. Data was captured overall on 19 control participants and 2 individuals with iSCI. For choosing a robust recognition model, five different classifiers have been trained, and validated via LOPOCV. Finally, a neural network model with 25 neurons has been chosen as the recognition model and was trained with $70\%$ of data from the 20 participants (18 controls, 2 individuals with iSCI). Overall accuracy for offline classification with 8 classes of activity on the test data set ($30\%$, n = 20) with the neural network model was 92.01%. It was challenging to compare recognition results to other studies due to different experimental setup (number of sensors, sensor placements, and recognition rate) and recognition method (offline/real-time). However, results for classifying “Sit to Stand” and “Stand to Sit” can be compared with a similar study on real-time human motion recognition, where an accuracy of 73.48% and 78.84% in recognizing “Sit to Stand” and “Stand to Sit” in real-time could be achieved, respectively [46]. In our recognition model, these values reached 90.98% and 90.42%.

Moreover, for activity recognition, various values for the window size, which defines the recognition rate and has an influence on accuracy, have been used in literature. The window size ranges from 3.88 s with 50% overlap [47] to 2 s with 1 s overlap [42]. Some smaller windows with a size of 1 s without overlap and 1.3 s with 50% overlap have also been used in recent years [32, 48]. To achieve a fast movement onset recognition, a sliding window with a fixed size of 100 ms with 90% overlap has been chosen in this work. The number of sensors and where to place them can be defined based on the activities which needed to be recognized. For instance, an important part of gait-related tasks is standing up and sitting down and, since the initiation of these transitions starts primarily through motion of the upper-body, an IMU was fixed on the sternum. Moreover, two other IMUs were placed on the middle of outer thighs of each participant to discriminate other tasks that have phasic or aphasic movements of the thighs.

Providing input for the assistive devices requires real-time data acquisition and analysis. We developed the neural network recognition model based on offline data analysis for later use in real-time scenarios in combination with gait rehabilitation devices. The developed model has been implemented in the embedded PC and tested on a participant. As Fig. 9 presents, the confusion matrices for offline and real-time classification on the validation data set, both had practically the same performance.

Not only robust performance in real time is necessary for providing input to the assistive device, but also the accuracy and safety are critical issues that needed to be considered. Figure 7 indicates where false recognitions occurred on streamed data for a subject when performing different activities. Data indicated that false recognitions usually occur in the transition phase between classes of activities like “Sitting” and “Sit to Stand” or “Standing” and “M. Standing”. These kinds of wrong recognitions could be due to imprecise labelling when defining the “true activity” in transition between two activities or between similar activities like “Sitting” and “M. Sitting”, which are hard to differentiate in continuous streaming and recognition.

Since LOPOCV ensures that different subjects are used for training and testing, results achieved indicate what can be expected for new users. Figure 6 showed the performance of the neural network model on 3 participants (a control and two individuals with iSCI) using LOPOCV. The classifier has still low performance on patient data (patient #2). In particular, the recognition model performed poorly in recognizing M.standing, Stand to Sit, and M.sitting. The first reason is that severely affected iSCI participants perform tasks slowly and 100 ms window size is small for detecting slow value changes. Second, the iSCI participant #2 was not able to perform tasks without walking aids such as standing upright. Therefore, the participant performed additional movements like grabbing/putting the crutches from/on the floor every time he/she was performing standing up/sitting down. Since these kinds of movements were not included in the model, which was trained mainly with control participants, recognising performances correctly that have not be seen before were difficult. Looking deeper into the differences in the feature vector between control and iSCI participants showed differences in the mean of the gyroscope signal of the sensor placed on the sternum and mean of left/right orientation of chest and thighs due to the mentioned additional movements. However, our goal is to obtain a robust algorithm on the long term that can capture movement performance ranging from impaired to healthy and allows also taking compensatory movements into account. Therefore, given the described limitations, the obtained results indicate the strong potential for the approach to work also in iSCI patients, when the algorithm can be trained with more additional patient data. Considering the slow pace of individuals with iSCI compared to controls while performing different tasks, the time window could be adjusted to a bigger time window in future. Furthermore, to take precautions, before selecting and applying a task-dependent support, a certain amount of time should be considered to recognize the same class several times in a row to avoid wrong recognition due to transitions and similarity between motions. The time can be estimated based on the needed time for the recognition model to reach a certain level of confidence.

## Conclusion and future work

In this work, a recognition model was designed for identifying the onset of most common activities in gait rehabilitation in real-time. The model was designed offline, based on data from three commercial inertial measurement units acquired from 18 control participants and 2 individuals with spinal cord injury. Inertial measurement units were attached to the sternum, and middle of outer thighs of the participants. Subsequently, the participants were asked to perform certain activities like sitting down, standing up, walking, and turning continuously with their preferred pace. Data from the inertial measurement units were streamed out via Bluetooth protocol into the designed wireless interface boards and then transferred to an embedded PC, which performed pre-processing and activity recognition in real time. Logged data was used to compare the performance of five different classification methods. The neural network model with 25 neurons was selected as a recognition model due to robust performance during Leave-One-Participant-Out Cross-Validation. Subsequently, the selected model has been trained with $70\%$ of the complete data set (n = 20) and tested, which yielded on all over performance of $92.01\%$ in F1-score. Thereafter, the final model was used for real-time activity recognition. Lastly, the performance of real-time classification was compared with offline classification on the data of one additional control participant. There was hardly any difference in performance between real-time classification ($88.6\%$) and offline classification ($88.68\%$) in F1-score. In this paper, our real-time classification and movement onset recognition was successfully tested and feasibility for a real-time implementation was shown. Accordingly, in future, we will implement our algorithm directly on a robot and test how the direct haptic interaction between robot and human will influence the human’s behaviour in a closed-loop setting.

## Supplementary Information

**Additional file 1: Table S1.** Extracted and chosen feature sets for recognition model generation. **Table S2.** Grid search results for neurons selection for different time windows and overlap rates as afunction of accuracy. **Figure S1.** Confusion matrices for all controls and two individuals with iSCI using LOPOCV.

## Data Availability

The data sets used and/or analysed during the current study are available from the corresponding author on reasonable request.

## Abbreviations

IMU: Inertial measurement unit
LOPOCV: Leave-One-Participant-Out Cross-Validation
DAQ: Data acquisition
RF: Radio frequency
SPI: Serial Peripheral Interface
UART: Universal Asynchronous Receiver-Transmitter
K-NN: K-Nearest Neighbour
SVM: Support Vector Machines
BWS: Body Weight Support
iSCI: Incomplete spinal cord injury
