# Supplementary material for: Real-time motion onset recognition for robot-assisted gait rehabilitation
Source: J Neuroeng Rehabil. 2022 Jan 28;19:11. doi: 10.1186/s12984-022-00984-x (PMC8796576; doi:10.1186/s12984-022-00984-x)
Supplement: Supplementary file 1 — Additional file 1: Table S1. Extracted and chosen feature sets for recognition model generation. Table S2. Grid search results for neurons selection for different time windows and overlap rates as afunction of accuracy. Figure S1. Confusion matrices for all controls and two individuals with iSCI using LOPOCV. [file 12984_2022_984_MOESM1_ESM.pdf]

Additional Materials

Table S1 Extracted and chosen Feature sets for recognition model generation

Number	Extracted features	Selected features
1	mean $(Angularvelocity_x - chest)$	✓Feature #1
2	mean $(Angularvelocity_y - chest)$	✓Feature #2
3	mean $(Angularvelocity_z - chest)$	×
4	std[a]$(Angularvelocity_x - chest)$	✓Feature #3
5	std $(Angularvelocity_y - chest)$	✓Feature #4
6	std $(Angularvelocity_z - chest)$	×
7	slope[b]$(Angularvelocity_x - chest)$	✓Feature #5
8	slope $(Angularvelocity_y - chest)$	×
9	slope $(Angularvelocity_z - chest)$	×
10	mean $(Acceleration_x - chest)$	×
11	mean $(Acceleration_y - chest)$	✓Feature #6
12	mean $(Acceleration_z - chest)$	✓Feature #7
13	std $(Acceleration_x - chest)$	✓Feature #8
14	std $(Acceleration_y - chest)$	✓Feature #9
15	std $(Acceleration_z - chest)$	✓Feature #10
16	slope $(Acceleration_x - chest)$	×
17	slope $(Acceleration_y - chest)$	×
18	slope $(Acceleration_z - chest)$	×
19	mean $(LinearAcceleration_chest)$	✓Feature #11
20	mean $(Angularvelocity_x - thighright)$	×
21	mean $(Angularvelocity_y - thighright)$	×
22	mean $(Angularvelocity_z - thighright)$	✓Feature #12
23	std $(Acceleration_x - thighright)$	×
24	std $(Acceleration_y - thighright)$	✓Feature #13
25	std $(Acceleration_z - thighright)$	✓Feature #14
26	slope $(Acceleration_x - thighright)$	×
27	slope $(Acceleration_y - thighright)$	×
28	slope $(Acceleration_z - thighright)$	×
29	mean $(Acceleration_x - thighright)$	×
30	mean $(Acceleration_y - thighright)$	✓Feature #15
31	mean $(Acceleration_z - thighright)$	×
32	mean $(Angularvelocity_x - thighleft)$	✓Feature #16
33	mean $(Angularvelocity_y - thighleft)$	×
34	mean $(Angularvelocity_z - thighleft)$	✓Feature #17
35	mean $(Acceleration_x - thighleft)$	×
36	mean $(Acceleration_y - thighleft)$	×
37	mean $(Acceleration_z - thighleft)$	✓Feature #18
38	std $(Acceleration_z - thighleft)$	×
39	std $(Acceleration_x - thighleft)$	✓Feature #19
40	std $(Acceleration_y - thighleft)$	✓Feature #20
41	slope $(Acceleration_x - thighleft)$	×
42	slope $(Acceleration_y - thighleft)$	×
43	slope $(Acceleration_z - thighleft)$	×
44	mean (Forward / backward orientation-chest)	×
45	mean (Left / right orientation-chest)	✓Feature #21
46	mean (Left / right orientation-thigh right)	✓Feature #22
47	mean (Forward / backward orientation-thigh right)	✓Feature #23
48	mean (Forward / backward orientation-thigh left)	×
49	mean (Left / right orientation-thigh left)	×
50	std (Forward / backward orientation-chest)	✓Feature #24
51	std (Left / right orientation-chest)	✓Feature #25
52	std (Left / right orientation-thigh right)	✓Feature #26
53	std (Forward/backward orientation-thigh right)	✓Feature #27
54	std (Forward/backward orientation-thigh left)	×
55	std (Left/right orientation-thigh left)	×

[a] Standard deviation
[b] Slope of fitted line on the data in a time window

Table S2 Grid search results for neurons selection for different time windows and overlap rates as a function of accuracy

Window size (sec)	Overlap (%)	Number of neurons	Accuracy
	90	23	0.90
80	70	26	0.91
	50	25	0.92
	90	25	0.92
100	70	21	0.92
	50	23	0.92
	90	25	0.92
160	70	26	0.93
	50	26	0.93
	90	25	0.93
200	70	23	0.93
	50	22	0.94

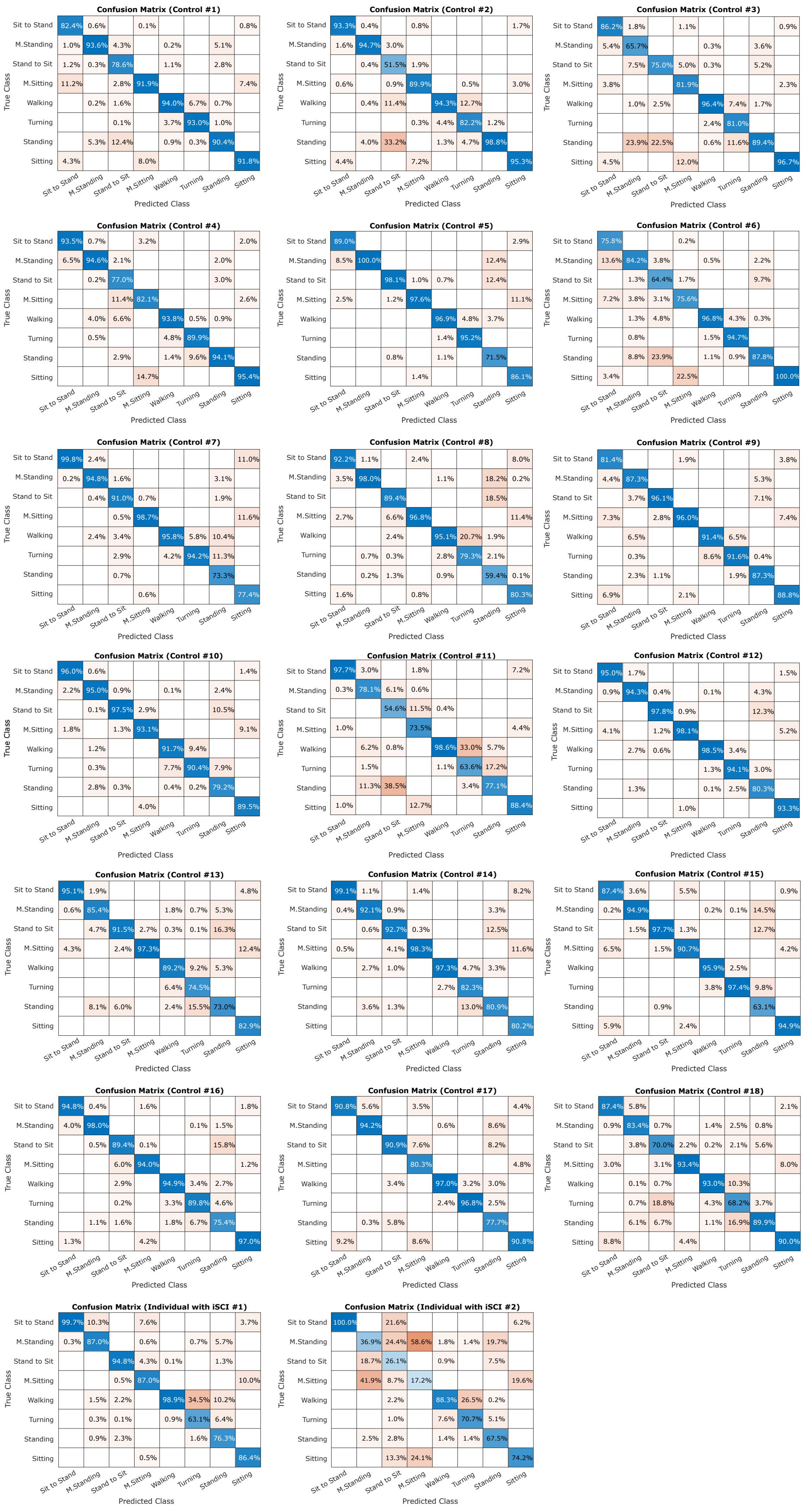

Figure S1 Confusion matrices for all controls and two individuals with iSCI using LOPOCV
